# Human saliva exerts strong type-dependent effects on adenovirus infectivity

**DOI:** 10.3389/fimmu.2025.1579896

**Published:** 2025-06-11

**Authors:** Erwan Sallard, Wenli Zhang, Nikita Chilakamarri, Setareh Farzanehkari, Inga Marte Charlott Seuthe, Anja Ehrhardt, Malik Aydin

**Affiliations:** ^1^ Virology and Microbiology, Center for Biomedical Education and Research (ZBAF), School of Medicine, Faculty of Health, Witten/Herdecke University, Witten, Germany; ^2^ Laboratory of Experimental Pediatric Pneumology and Allergology, Faculty of Health, Witten/Herdecke University, Witten, Germany; ^3^ Department of Otorhinolaryngology, Head and Neck Surgery, University of Witten/Herdecke, Catholic Hospital Hagen, Hagen, Germany

**Keywords:** human adenovirus, saliva, oral vaccine, mucosal immunity, mucosal delivery, epithelium infection

## Abstract

**Background:**

The development of mucosal adenovirus (Ad) vaccine vectors is considered one of the next frontiers to protect vulnerable patients from respiratory and gastrointestinal pathogens. An efficient delivery to or through the oral cavity necessitates a thorough understanding of Ad interactions with saliva for oral, buccal or sublingual vaccine delivery, which could additionally prove instrumental in the containment of natural Ad infections but remains unexplored. Therefore, we investigated the influence of saliva on Ad infectivity, emphasizing its intrinsic antiviral role against particular Ad types in various epithelial cell cultures.

**Methods:**

A saliva pool was created from healthy donors (*n*=16) and incubated with ChAdOx1 or human Ads from 20 different types prior to infection of human immortalized epithelial cells. All human Ads used were replication-competent and expressed a GLN cassette containing a green-fluorescent protein, nano-luciferase, and neomycin resistance. Loss-of-function experiments were conducted by immunoprecipitation or enzymatic digestion of specific saliva components to decipher related mechanisms.

**Results:**

Temporal and inter-individual variability in saliva samples were observed, validating the use of a saliva pool to represent the population. Saliva strongly influenced Ad infectivity, in general through inhibiting species B types and enhancing species D and E Ads, that include the vaccine vector platforms Ad26 and ChAdOx1. Interestingly, Ad20 presented the highest infectivity enhancement, as well as superior to average salivary mucus crossing rates. Furthermore, saliva immunoglobulins and human neutrophil peptides marginally influenced the Ad infectivity, while sialic acid inhibited all tested Ad types.

**Conclusion:**

Saliva may have a protective role against infection by certain types of Ads. This discovery highlights a potential limitation in the efficacy of next-generation oral Ad vaccine vectors. Consequently, our study underscores the importance of identifying and utilizing saliva-resistant Ad vectors to optimize Ad-based vaccination strategies.

## 1 Introduction

Adenoviruses (Ad) are double-stranded DNA viruses with a genome of 26 to 48 kb. There are more than 300 known mammalian Ad types, including 116 currently known human Ad types grouped in seven species (A to G) (1) that differ in clinically relevant features including seroprevalence or tropism. Adenoviruses are a frequent cause of mild respiratory infections (2), associating with up to 15% of upper respiratory tract infections, but also of more severe symptoms including acute gastroenteritis and pharyngo-conjunctival fever, which is frequently attributed to species B Ad including human Ad type 3 (Ad3) (3). On the other hand, numerous human and simian Ad types have been genetically modified as vectors for gene therapy, oncolytic virotherapy or vaccination. In particular, several Ad vector vaccines have been approved against SARS-CoV-2, few of them being administered intramuscularly (notably the so-called ‘*AstraZeneca’* and ‘*Janssen’* vaccines, derived from the ChAdOx1 and Ad26 platforms, respectively), while others were designed for intranasal (including the iNCOVACC vaccine (4)) or orally inhaled application (for example, the Convidecia vaccine (5)).

Mucosal vaccines are indeed in increasingly high demand, with the main purpose of stimulating strong and long-lasting local immunity at the site of infection of the target pathogen in addition to systemic immunity. Most currently approved mucosal vaccines are delivered intranasally or orally to immunize the intestine (6), while the buccal cavity also appears to be a promising target (7). However, mucosal vaccination still faces numerous challenges including the need to adapt vaccine components to complex and variable mucosal environments. In detail, successful immunization requires resisting to mucosal secretions, crossing mucus and epithelial layers, and then inducing a response of desired strength and type from the mucosa-associated lymphoid tissue. In case of viral vectors administered to or through the oral cavity for mucosal vaccination or oncolytic treatment, clinical success may thus rely on the conservation of infectivity in saliva.

Saliva is a complex fluid whose composition may vary substantially between individuals, as well as across time based on hydration, diet or infection history (8). In addition, saliva plays a major role not only in digestion, but also in microbiome control and protection against pathogens. It contains immunoglobulins (Ig), among which the IgA class is the most abundant, while IgG and IgM concentrations in saliva usually are one or two orders of magnitude below (9). Furthermore, saliva contains numerous antimicrobial peptides, including the α-defensins human neutrophil peptides (HNP) 1, 2, 3 (10), and the substantially less abundant HNP-4 (11). Purified HNP-1 was reported *in vitro* to increase the infectivity of species D Ad in epithelial cells but decreases that of species B or C Ad (12–14). Likewise, sialic acid, which is a common glycosylation type displayed on numerous salivary proteins and lipids, as well as in serum and in tissues, was shown to inhibit Ad5 infectivity when present in the extracellular matrix of the epithelial cells (15, 16), whereas it is used by most species D Ad as a receptor (17). Saliva is also able to inactivate viruses including HIV-1 (18), SARS-CoV-2 (with a positive correlation with saliva specific IgA levels (19)), and influenza A virus (20). However, the influence of saliva on human Ad has to our knowledge not been studied yet.

Here, we study the interactions of saliva with a wide range of Ad and investigate the influence of various components of saliva, including Ig, defensins, and sialic acids, on Ad infectivity.

## 2 Materials and methods

### 2.1 Cell culture

For cell culture work, we used an immortalized cell line (lung adenocarcinoma-derived A549 lung cells), which was cultivated in Modified Eagle Medium (MEM, Pan-Biotech) with 10% Fetal Bovine Serum (FBS) (Pan-Biotech), and Penicillin/Streptomycin (P/S, Pan-Biotech). In addition, 93VU147T head-and-neck cancer cells (hereafter termed HNC) were cultivated in Modified Eagle Medium (MEM) + 10% FBS + 2 mM L-Glutamine (Pan-Biotech) + P/S + non-essential amino acids (NEAA, Pan-Biotech #P08-32100). After infections were performed, the percentage of FBS in the culture medium was lowered to 2%. Cells were then cultivated at 37°C under an atmosphere with 5% CO_2_ and were confirmed free of *Mycoplasma* infection using the VenorGeM OneStep kit (Minerva Biolabs).

### 2.2 Adenovirus acquisition

Human Ad vectors expressing TurboGFP (GFP=green fluorescent protein), NanoLuc-luciferase, and the selection marker kanamycin/neomycin under a synthetic CAG promoter in their deleted E3 locus were already described previously (21, 22). The ChAdOx1 vector expressing eGFP under a CMV promoter in the deleted E1 locus was also described previously (23). The virus particle concentration of the adenovirus-GLN library was conducted by measuring the optical density at 260 nm via spectrophotometer and described as viral particles (vps) per milliliter (21, 22).

### 2.3 Saliva collection

Saliva was collected from healthy subjects (*n*=16: age range: 35.06 ± 9.57 years; 11 females and 5 males), all of whom provided informed consent. In addition, the subjects did not eat, drink or smoke for at least one hour before donation. Unless specified otherwise, saliva was cleared of large particles by centrifugation for 2 min at 10,000 x g, was kept at 4°C, and used on the same day.

### 2.4 Immunoglobulin and HNP depletion

Cleared saliva was incubated for 2 h at 4°C under gentle shaking with 2 µg/mL of mouse IgG1 anti-human IgA (Miltenyi Biotec, clone IS11-8E10), IgG (Miltenyi Biotec, clone IS11-3B2.2.3), IgM (Biolegend, #314519), HNP1-3 (Hycult Biotech, clone D21), or anti-HPV16/18 E6 (Santa Cruz, sc-460) as negative control. For 1 volume of saliva, 0.2 volumes of thoroughly homogenized magnetic beads (dynabeads pan-mouse IgG, Invitrogen, #11041) were washed twice with PBS then resuspended in antibody-treated saliva and incubated for 1 h at 4°C under gentle shaking. Treated saliva was then placed on magnets for > 2 min, and the supernatant was transferred to a new tube. The bead decantation was repeated.

For immunoblots to validate IgG depletion ***(*** **Supplementary Figure 1** ***)***, 15 µL of samples to be tested were denatured with Laemmli buffer and loaded on polyacrylamide gels for electrophoresis then blotted on a nitrocellulose membrane. The membrane was blocked in Odyssey blocking buffer (LI-COR Biosciences) for 1 h at room temperature (RT) and washed three times with TBS-Tween. Then, the membrane was incubated with goat anti-human IgG coupled with RD680 fluorophore (LI-COR Biosciences, #926-68078) diluted 1:10000 in Intercept T20 antibody diluent (LI-COR Biosciences) for 90 min at RT. The membrane was washed three times, dried and fluorescence emitted at 700 nm was imaged using an Odyssey CLx imaging system (LI-COR Biosciences). The relative quantity of target proteins was estimated by measuring the integrated fluorescence intensity at the corresponding spot after background subtraction using Fiji (ImageJ), which is an open-source platform for image analysis.

### 2.5 Infectivity assays

1.1E7 vector particles (VP) of Ad were incubated for 1 h at 37°C in 44 µL of cleared saliva having received the desired treatment or of optiMEM (Gibco) as a control group. Suspensions were then mixed with 396 µL of pre-warmed optiMEM + P/S and 100 µL were distributed per well of confluent target cells grown in 96 well plates, corresponding to 50 VP per cell. At 3 hours post infection (hpi), the infection mix was replaced with culture media.

At 24 hpi, luciferase luminescence was quantified on a TECAN infinite f plex plate reader (TECAN) using black 96-well luciferase plates (Thermo Fisher Scientific Nunc A/S) and the Nano-Glo^®^ Luciferase Assay kit (Promega, Madison, USA) following the manufacturer’s protocol. Alternatively, GFP-positive cells were counted at 48 hpi by flow cytometry (CytoFlex, Beckman Coulter, Munich, Germany) using the FITC channel (585/42 nm), excited with a 488 nm laser.

To validate the use of reporter genes in infectivity assay ***(*** **Supplementary Figure 2** ***)***, we tested the proportionality of luminescence levels with infectious dose for a given Ad type by infecting A549 cells with 2, 20, 200 or 2000 VP per cell of Ad5 or Ad35 and then quantified luminescence as described above.

For sialic acid depletion assays, cleared saliva was treated with 5 mU sialidase (Roche #10269611001) at 37°C for 30 min before incubation with Ad VPs or left untreated. After VP incubation for 1 h at 37°C, nine volumes of optiMEM + P/S + 11 µg/mL oseltamivir (to inactivate sialidase and avoid saliva-independent effects of sialidase on target cells) were added and distributed onto target cells, corresponding to 50 VP per cell. The cell culture media was then changed at 3 hpi, and luciferase luminescence was quantified at 24 hpi as described above.

### 2.6 Mucus crossing assays

For mucus crossing assays, 50 µL of whole saliva were deposited per tissue culture insert (PET-coated, 1 µm pores, Sarstedt #83.3932.101) and left to dry for 16 h at 37°C. Meanwhile, 24-well tissue culture plates were coated with PBS + 2% gelatin (Sigma #G1890). The liquid phase was then removed from the wells, which were left to dry at RT for 1 h. 200µL PBS were added in each well (lower compartment), then a mucus-coated or uncoated insert, in which were added 1E9 Ad VPs in 80 µL H2O + 20 µL cleared saliva from the population pool. Following 3 h incubation at 37°C, the lower compartment was collected, mixed with Tris-EDTA (TE) buffer + 0.5 µg/mL proteinase K + 5mM EDTA + 0.5% SDS and incubated for 3 h at 56°C with gentle shaking to release Ad genomes from their capsids. DNA was then purified by ethanol precipitation, resuspended in water and mucus-crossing VPs were quantified by qPCR using the primers TGCTCCTGCCGAGAAAGTAT and GCTCTTCGTCCAGATCATC, a CFX96 Real-Time System machine (BioRad) and the my-Budget 5x EvaGreen qPCR-Mix II (Bio-Budget) following the manufacturer’s instructions.

### 2.7 Neutralizing antibodies

Human sera were collected in serum tubes (Sarstedt 01.1601) from healthy volunteers, heated at 56°C for 30 min and diluted 1:10 in pure MEM. Two-fold dilution series up to a serum dilution of 1:2560 were then performed using MEM + 10% FBS as diluent in order to equalize the total serum concentration, then incubated for 1 h at 37°C with 5 E7 VP/mL of Ad5. As a control, MEM + 10% FBS was used. The incubation mix was then distributed on confluent A549 cells, resulting in 100 VP/cell (vpc), the media was changed at 3 hpi, and luciferase luminescence was measured at 24 hpi as indicated above.

### 2.8 Statistical analyses

The statistical analyses were performed with R. Error bars represent the standard deviation. Statistical tests were computed on R using the libraries readxl, rstatix and DescTools. The significance level was set at *p* < 0.05. Certain figures were created using BioRender. The number of biological replicates (*n*) is indicated in the figure legend for each experiment.

### 2.9 Ethics approval

The study was approved by the ethics committee of the Witten/Herdecke University, Germany (approval number S-342/2023; January, 4^th^ 2024, amendment June 17^th^ 2024). The study and the analyses were in accordance with the ethical standards, the 1964 Helsinki declaration, and its later amendments. All donors gave informed consent prior biomaterial collection.

## 3 Results

### 3.1 Human saliva inactivates certain adenovirus types but enhances the infectivity of other types

**Figure 1:**
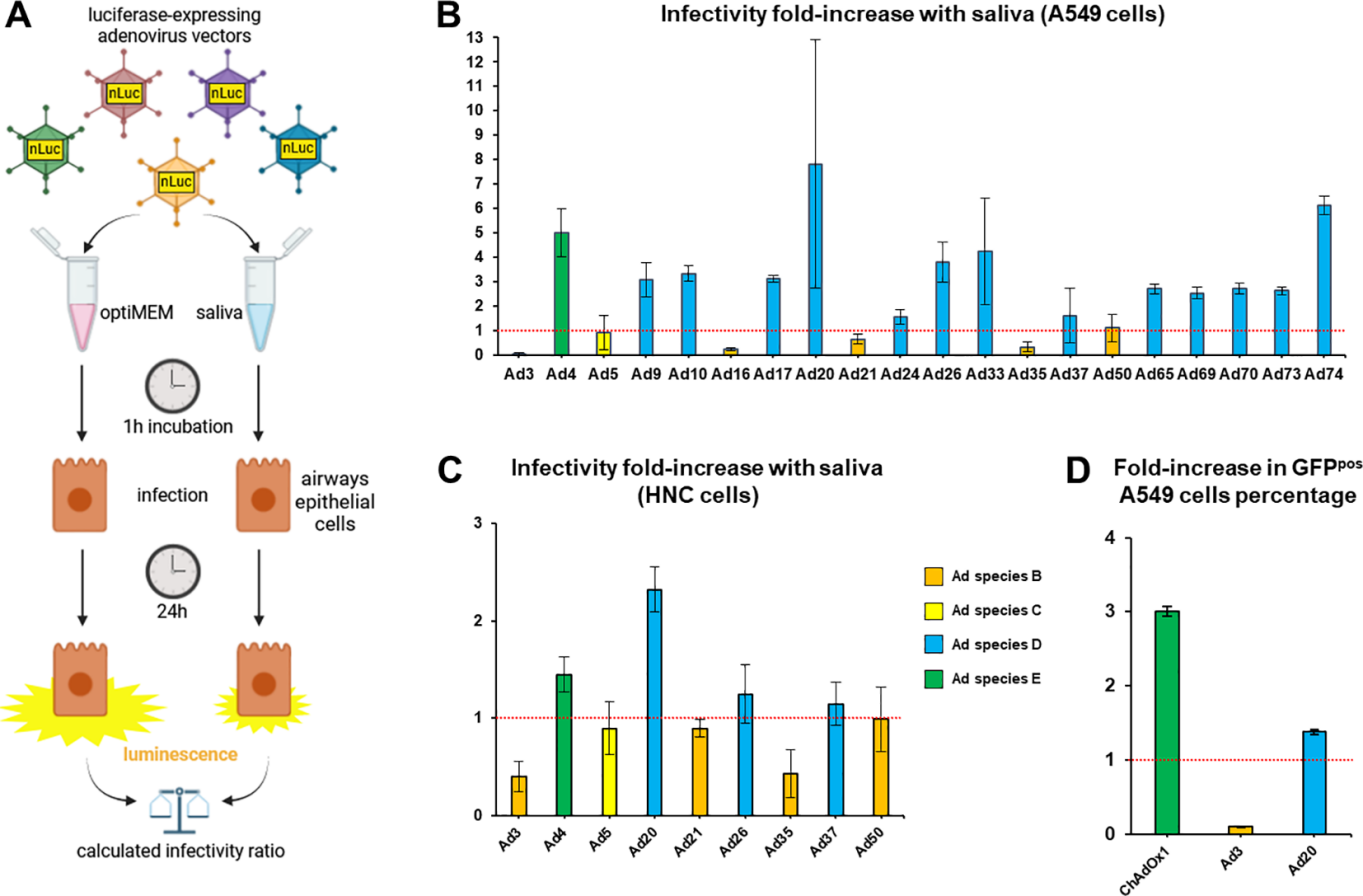
A saliva pool influences the infectivity of an adenovirus collection. **(A)** Experimental design. Adenovirus vectors were incubated in a pool of cleared saliva collected from *n*=16 healthy donors or in optiMEM, then allowed to infect A549 **(B)** or HNC **(C)** cells. Luminescence levels measured 24 hours post infection (hpi) in saliva-treated groups were normalized on levels from the cognate optiMEM control groups to determine the effect of saliva on the infectivity of each adenovirus type (infectivity fold-increase). **(B)** Adenovirus collection screen in lung carcinoma A549 cells, *n*=4 to *n*=12, one to three independent repeats. **(C)** Screen of select adenovirus vectors in head-and-neck carcinoma cells (HNC) (*n*=4). **(D)** GFP-expressing ChAdOx1, Ad3 and Ad20 vectors were incubated in a pool of cleared saliva collected from *n*=16 healthy donors or in optiMEM, then allowed to infect A549 cells. The percentages of GFP-positive cells at 48 hpi in saliva-treated groups were normalized on percentages from the cognate optiMEM control groups (*n=4*).

We created a pool of saliva collected from healthy volunteers (*n*=16) as a model of population-scale saliva composition. At first, we studied cleared saliva that had been centrifuged to eliminate large particles in order to investigate biochemical effects of saliva on virus particles rather than trapping in the mucus or the influence of the microbiome. We measured the effect of saliva, as compared to serum-free medium, on the infectivity of Ad drawn from a collection of luciferase expressing vectors comprising representatives of human Ad species B, C, D, and E (**Figure 1A**). In A549 cells, saliva led to nearly complete inactivation of Ad3, to an extensive inhibition of Ad16, Ad21 and Ad35, but to an enhancement of several Ad types, in particular Ad4, Ad20 and Ad74 (**Figure 1B**). Moreover, testing the effect of saliva on a select group of Ad types in head-and-neck carcinoma (HNC) cells, saliva displayed similar effects in HNC cells compared to A549 cells, although slightly attenuated (**Figure 1C**). Of note, HNC cells are closer representatives of cells directly exposed to saliva *in vivo* than the pulmonary A549 cells due to their laryngopharyngeal origin. Finally, we extended our screening to GFP-expressing ChAdOx1, Ad3 and Ad20 vectors in A549 cells, observing as before a nearly complete inactivation of Ad3, while Ad20 and ChAdOx1 showed enhanced infectivity in presence of saliva (**Figure 1D**).

**Figure 2:**
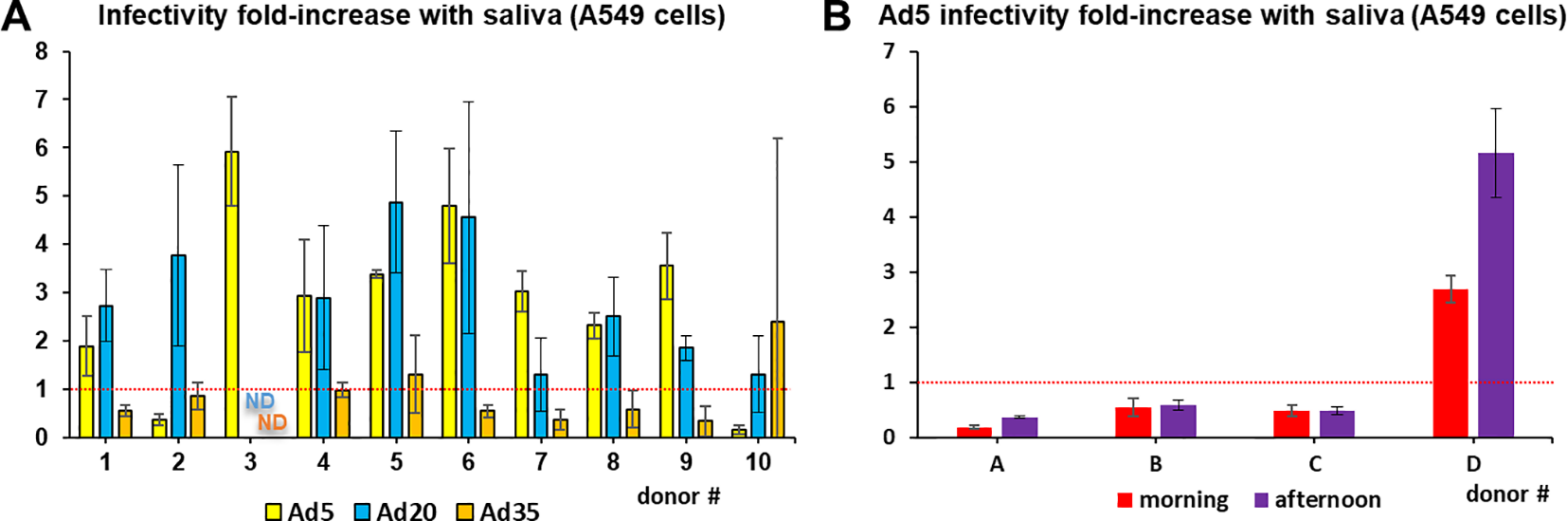
Inter-individual and temporal variability in saliva effect on adenoviruses. **(A)** Infectivity assays in A549 cells were conducted with Ad5, Ad20 and Ad35 using cleared saliva from individual donors (*n*=4). ND=no data **(B)** Ad5 infectivity assays in A549 cells were conducted using cleared saliva from individual donors collected the same day at 9 am or 1 pm (*n*=4).

However, individual saliva samples revealed substantial variability in their effects on Ad infectivity. For example, Ad5, whose infectivity was hardly affected by incubation with the saliva pool model, showed up to six-fold enhancement with certain individual saliva samples and up to seven-fold inhibition in others (**Figure 2A**). Variability was even observed in saliva samples collected from the same donors at 4 h intervals (**Figure 2B**).

### 3.2 Immunoglobulins and HNP exert minor to no effects on adenovirus infectivity

**Figure 3:**
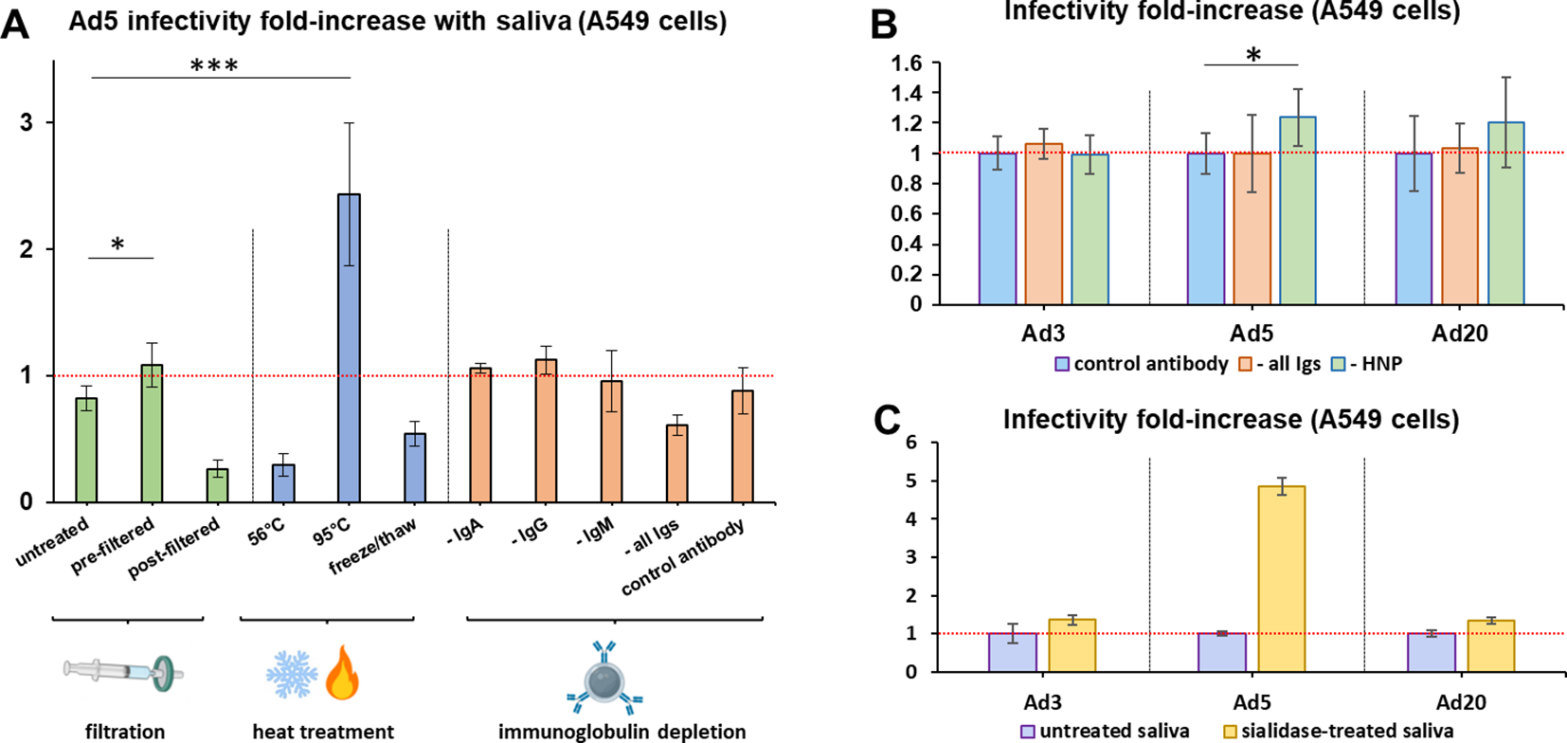
Sialic acid and partially heat-resistant compounds, but not immunoglobulins, decrease adenovirus infectivity in saliva. **(A)**. Ad5 infectivity assays in A549 cells were conducted using the cleared saliva pool without further treatment (‘untreated’), or that had been filtered before (‘pre-filtered’) or after (‘post-filtered’) incubation with Ad5 using 0.45 µm filters, or heat-treated for 30 min at 56°C or 95°C, or frozen and thawed three times, or depleted for immunoglobulins A (‘- IgA’), G (‘- IgG’), M (‘- IgM’) or all three immunoglobulin classes (‘- all Igs’), or submitted to the depletion protocol using an irrelevant anti-papillomavirus antibody as control (‘control antibody’). *n*=7 to *n*=19, two to five independent repeats. Following Welch’s ANOVA test of all samples (*p*=8.88E-11), *post-hoc* Dunnett tests were conducted using untreated as control group. Significant differences were found only for the ‘pre-filtered’ and ‘95°C’ groups. *,0.01<*p*<0.05; ***, *p*<0.001. **(B)**. Infectivity assays in A549 cells were conducted with Ad3, Ad5 and Ad20 using the cleared saliva pool depleted for IgA, IgG and IgM (‘- all Igs’); or for HNP1-3 (‘- HNP’); or using the control anti-papillomavirus antibody (‘control antibody’), *n*=8, two independent repeats. Infectivity levels were normalized on the control group for each virus. Comparisons were conducted between the control group and either ‘- all Igs’ or ‘- HNP’ for each virus using Mann-Whitney U and T-tests, that both found significant differences with the control group only for Ad5 and HNP depletion. *, 0.01<*p*<0.05. **(C)** Infectivity assays in A549 cells were conducted with Ad3, Ad5 and Ad20 using the cleared saliva pool depleted of sialic acid by sialidase digestion (*n*=3). Infectivity levels were normalized on the control group for each virus.

In order to understand which components of saliva may influence Ad infectivity, loss-of-function assays on the cleared saliva pool model were performed. Since 0.45 µm filtration of cleared saliva is known to eliminate a fraction of aggregates and high-molecular weight components, including mucins and agglutinins, saliva was filtered before or after incubation with Ad5, leading respectively to an increase and a decrease in infectivity (**Figure 3A**). Likewise, inactivation of heat-labile compounds at 95°C significantly increased infectivity, while moderate heating (56°C) and freezing tended to decrease it.

Furthermore, we selectively depleted various classes of Ig by co-immunoprecipitation, leading to the removal of >99% targeted proteins as validated in the case of IgG (**Supplementary Figure 1**). However, depletion of IgA, IgG, IgM, or all three classes simultaneously from saliva did not affect Ad5 infectivity (**Figure 3A**). This result was also observed with the strongly saliva-inactivated Ad3 and the saliva-enhanced Ad20 (**Figure 3B**), whereas the major defensins HNP1–3 decreased Ad5 infectivity by only 19% and did not significantly influence Ad3 or Ad20.

We then tested the influence of salivary sialic acid, which is a well-known receptor of species D Ad, on Ad infectivity. Digestion by sialidase increased the infectivity of all tested Ad types (**Figure 3C**), irrespectively of whether they target sialic acid as a receptor (Ad20 does, Ad3 and Ad5 do not).

### 3.3 Salivary mucus hampers adenovirus diffusion

**Figure 4:**
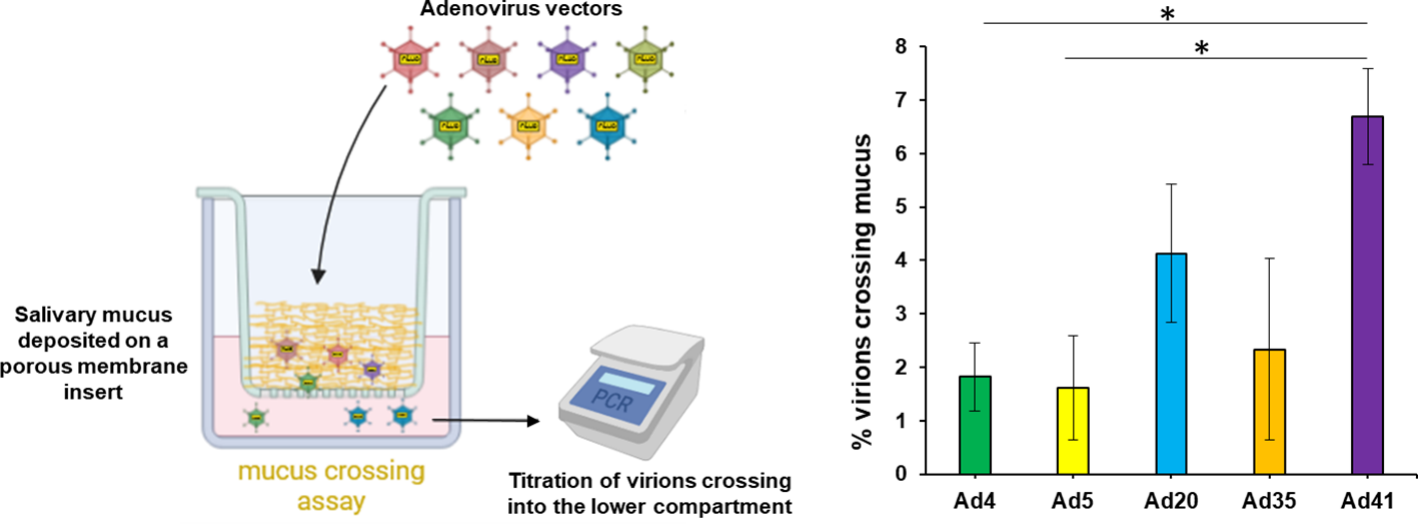
Salivary mucus hinders adenovirus diffusion with type dependency. To test the adenovirus diffusion through salivary mucus, Ad4, Ad5, Ad20, Ad35 and Ad41 virus particles were incubated in diluted cleared saliva pool in the upper compartment above a porous membrane coated or not with salivary mucus during 3h at 37°C. Virions crossing into the lower compartment (PBS) were quantified, *n*≥4, two independent repeats. Percentages of mucus-crossing virions were calculated for each adenovirus type by normalization of titers obtained with mucus-coated inserts on those with uncoated inserts. These percentages were compared between adenovirus types by Kruskal-Wallis test (*p*=0.007782) since a Shapiro-Wilk test indicated non-normal distribution, and pairwise comparisons were performed by *post-hoc* Dunn’s test with Holm’s correction. *,0.01<*p*<0.05. For normalization, no saliva was used as controls and the titers were normalized on non-mucus transwells. Due to the origin of the purchased transwell inserts, PET cannot be removed from the transwells.

Besides its fluid phase, saliva contains a solid phase primarily composed of high molecular weight mucins and agglutinins. To study its effect on Ad, we dried whole saliva on porous membranes, re-humidified it and tested the ability of several Ad types to diffuse through the mucus layer. The enteric-tropic Ad41 displayed the highest mucus-crossing ability, followed by Ad20, above Ad4, Ad5 and Ad35 for which only around 2% of virus particles were able to cross the mucus layer (**Figure 4**).

**Table 1** summarizes the effects of native and sialidase-treated saliva on the different Ad types, as well as their mucus crossing levels, and indicates the tropism and receptor usage of each of the Ad types evaluated.

**Table 1:** Comparison of the effect of saliva and sialidase treatment of saliva on adenovirus infectivity [articles and reviews in (22, 24–33)].

Adenovirus type	Adenovirus species	Tropism	Effect of saliva	Effect of Sialidase treatment of saliva	Mucus crossing ability	Receptors
Ad3	B	Airway, Eye	↓↓ Inhibition	↑ Enhancement		CD46, CAR, HSPG, DSG-2
Ad4	E	Airway, Eye	↑↑ Enhancement		weak (1.8%)	CAR
Ad5	C	Airway, Eye, Liver	≈ Stable	↑ Enhancement	weak (1.6%)	CAR, HSPG, Integrins
Ad9	D	Eye	↑↑ Enhancement			CAR, CD46, Sialic Acid
Ad10	D	Eye	↑↑ Enhancement			CAR, CD46, Sialic Acid
Ad16	B	Airway, Eye	↓↓ Inhibition			i.e., CD46, HSPG, CAR
Ad17	D	Eye	↑↑ Enhancement			CAR, CD46, Sialic Acid
Ad20	D	Eye	↑↑ Enhancement	↑ Enhancement	intermediate (4.1%)	CAR, CD46, Sialic Acid
Ad21	B	Airway, Eye	↓↓ Inhibition			i.e., CD46, HSPG
Ad24	D	Eye	↑↑ Enhancement			CAR, CD46, Sialic Acid
Ad26	D	Eye	↑ Enhancement			CAR, CD46, Sialic Acid
Ad33	D	Eye	↑↑ Enhancement			CAR, CD46, Sialic Acid
Ad35	B	Airway, Eye, Genito-/ urinary tract	↓↓ Inhibition		weak (2.3%)	CD46, Integrins, HSPG
Ad37	D	Eye	≈ Stable			CAR, CD46, Sialic Acid
Ad50	B	Airway Eye	≈ Stable			i.e., CD46, HSPG
Ad65	D	Eye	↑ Enhancement			*Not known* *Most probably:* e.g., CD46, Sialic Acid
Ad69	D	Eye	↑ Enhancement			CAR
Ad70	D	Eye	↑ Enhancement			CD46
Ad73	D	Eye	↑ Enhancement			CD46
Ad74	D	Eye	↑↑ Enhancement			CD46, CAR
Ad41	F	GIT			strong (6.7%)	CAR

(CAR, Coxsackie and adenovirus receptor; HSPG, Heparan sulfate proteoglycan; DSG2, Desmoglein 2; GIT, gastrointestinal tract).

Green: increase in Ad infectivity or strong mucus crossing; Blue: no significant effect on Ad infectivity or moderate mucus crossing; Purple: decrease in Ad infectivity or weak mucus crossing levels.

## 4 Discussion

Here we studied for the first time the interactions of saliva with a broad range of Ad from different species, showing an impact on infectivity consistent across airway epithelial cell lines and variable between Ad types (**Figure 1**). In particular, saliva enhanced the infectivity of all tested species E (Ad4 and ChAdOx1) and species D (Ad9, 10, 17, 20, 24, 26, 33, 37, 65, 69, 70, 73, and 74) Ad, but decreased the infectivity of all tested species B Ad (Ad3, 16, 21, and 35) except Ad50. Adenoviruses from species D and E are frequently associated with ocular and gastrointestinal infections and may exploit glycan-mediated interactions in mucosal environments. In contrast, Ad of species B, which are typically associated with respiratory and/or genitourinary/renal tropism may be more susceptible to inhibitory components in saliva. These hypotheses need to be further investigated in future studies.

Furthermore, this study illustrates the importance of taking into account mucosal environments when designing viral vectors for vaccination or cancer treatment. In particular, Ad3 was deemed a promising candidate for oral and respiratory delivery due to high infection levels observed in absence of saliva in HNC cells (34) or human primary airway epithelia (35), but was shown in our study to suffer near-complete inactivation in presence of saliva, compromising *in vivo*-applications. On the contrary, our screening adds arguments in favor of certain vectors including the vaccine platforms Ad26 and ChAdOx1 as well as Ad4, Ad20 and Ad74, whose transduction levels were enhanced by saliva. Although the observed inter-individual variability (**Figure 2**) warrants caution on vector choice, the fact that the infectivity of Ad20 was increased by the saliva of all tested donors (**Figure 2A**) raises hopes regarding the applicability of these vectors. The enhancement of Ad4 in presence of saliva possibly contributed to its successful use as a live vaccine vector with oral delivery in the American military (36) and to the improved anti-influenza hemagglutinin antibody induction by an Ad4 vaccine vector observed in the NCT01443936 clinical trial after oral administration compared to the intranasal route (37, 38). A recently described E1/E3-deleted Ad4 vector platform ready to incorporate transgenes for vaccination or gene therapy purposes (34) may contribute to additional applications.

Though thinner than in other compartments of the digestive and respiratory tracts, the buccal cavity is delineated by a mucus layer that may trap virus particles and prevent them to infect the underlying epithelium. Unfortunately, *in vitro* models of epithelium cultures with mucus are rare and their mucus layer is substantially thicker than *in vivo* (39), prompting us to rely on a physical diffusion test to estimate the effect of salivary mucus on Ad. Unlike Ad4, Ad20 displayed a relatively high mucus crossing rate (**Figure 4**), further supporting its potential applicability for mucosa transduction. In addition, Ad41 showed the highest mucus crossing rate among tested Ad, consistently with its enteric tropism which implies that it faces thick mucus layers during its natural life cycle.

Unexpectedly, saliva Ig did not significantly influence the infectivity of the tested Ad (**Figure 2**) despite the high seroprevalence of Ad3 and Ad5 (40). In particular, six of the donors who provided individual saliva samples were also tested for serum anti-Ad5 neutralizing antibodies and were all positive (data not shown), yet all but one (donor #2) of their saliva samples increased Ad5 infectivity. It is unlikely that the <1% Igs remaining after depletion would suffice to mediate the same effects as untreated saliva if strong Ig-mediated inactivation had occurred. However, our observations do not exclude that saliva Igs bind certain Ad without neutralizing them. Furthermore, HNPs had only limited effects on Ad5 infectivity and none on Ad3 and Ad20; the divergence with previous results may be explained by the high doses of >5 µM HNP-1 necessary to observe Ad5 inactivation in *in vitro*-studies (12–14), which is superior to its physiological range of 0.3-3µM in saliva (41). Finally, sialic acid decreased the infectivity of all tested Ad types (**Figure 3C**), consistently with its effect on Ad5 when present in the extracellular matrix, where sialic acid was reported to decrease Ad5 binding to target cells due to steric hindrance and electrostatic repulsion (15, 16). Salivary sialic acid may likewise limit Ad particles diffusion towards their cellular receptors, in addition to competing for fiber knob binding in the case of Ad types that use sialic acid as receptor.

Consequently, the saliva components responsible of the major infectivity enhancement of certain Ad types could not be identified in this study. It can however be proposed that aggregates reversibly trap and block part of Ad particles, as suggested by the influence of filtration on infectivity, and consistently with the mechanisms of HIV-1 inactivation by saliva (18). The respective infectivity decrease and increase in saliva treated at 56°C or -20°C on the one hand, and 95°C on the other, may suggest that labile proteins other than Ig and defensins increase Ad infectivity while more structurally stable ones decrease it. However, this could also be attributed to changes in the physico-chemical environment in saliva driven, for example by viscosity changes in case of temperature variation. Microfluidics and diffusion studies may in the future help characterize the reaction of Ad particles to changes in saliva properties.

Saliva contains several proteins, enzymes, and may show antimicrobial role (20, 42, 43). Salivary amylase (alpha-amylase) is highly abundant in human saliva and is secreted by salivary glands (20, 44–46). The substrates of salivary amylase are among others starch and glycogens, which amylase degrades by specifically cleaving alpha-1,4-glycosidic bonds (reviewed in (45–47)). Furthermore, salivary amylase is not a protease (reviewed in (43, 45–47)) and it does not break peptide bonds and therefore should not have effects on viral capsid proteins. White and colleagues showed that contrary to salivary proteins and glycoproteins including MUC5B and defensins, amylase had no inhibitory impact on influenza A virus (20). As Ads are protein-based, non-enveloped viruses whose capsid proteins are scarcely glycosylated and do not carry starch-like carbohydrates, amylase could not be expected to influence Ad infectivity and was not investigated here.

Our study presents certain limitations. First, anti-Ad5 serum antibodies were titrated for only six donors. Our depletion experiments indicate that they do not play a relevant role. However, materials to quantify the anti-Ad-IgA and –IgG titers in saliva were not available and our results may not apply to specific individuals with particularly high titers. Anti-Ad serum antibody titers and seroprevalence have been investigated in multiple cohorts (48, 49), but to our knowledge no data on anti-Ad salivary titers has been published to date. Although our results indicate that relatively high serum anti-Ad5 titers do not translate into salivary inhibition, this conclusion may not apply to all Ad types.

Second, we focused our study on the effects of saliva on Ad productive infection levels as indicated by reporter gene expression at early time points (**Supplementary Figure 2**), which may not be perfectly correlated with vector binding or replication levels and other stages of viral life cycle. Third, since all donors were young or middle age adults, our findings may not be generalizable to pediatric and geriatric populations and this has to be considered in further studies. In addition, *in vivo*-validation using animal models, or including recently vaccinated or infected donors, would represent valuable additions to this study. Furthermore, high-throughput correlation studies linking the microbiota composition or saliva proteome to Ad infectivity could improve the mechanistic understanding of inter-individual variability. In addition, pooled saliva may mask individual variability, despite attempts to address this through longitudinal or temporal sampling strategies.

Besides its effects on Ad infectivity, saliva may enhance or dampen the antiviral immune response and thus mediate broader effects on adenoviral infection or treatment course than merely through infectivity effects. Since the oral way is not the only route of Ad infection nor the only one considered for Ad vector mucosal administration, the influence of other secretions (nasal secretions, tears) may also be studied. Nevertheless, saliva may also be expected to be active in non-buccal compartments and in particular the pharynx and larynx along the digestive tract.

## Data Availability

The raw data supporting the conclusions of this article will be made available by the authors, without undue reservation.
