# Supplementary material for: Human saliva exerts strong type-dependent effects on adenovirus infectivity
Source: Front Immunol. 2025 Jun 11;16:1579896. doi: 10.3389/fimmu.2025.1579896 (PMC12188253; doi:10.3389/fimmu.2025.1579896)
Supplement: Supplementary file 1 [file DataSheet1.docx]

**Supplementary material**


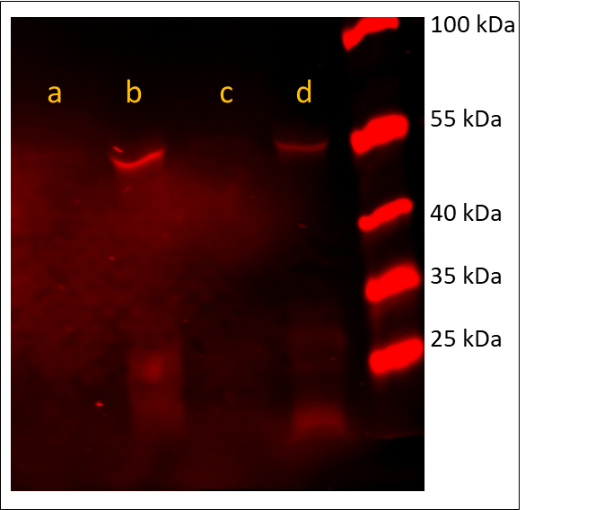


**Supplementary Figure 1. Immunoblot validation of the depletion protocol.** The IgG depletion protocol was performed twice independently using the cleared saliva pool. 15µL of undiluted IgG-depleted saliva (a, c) and 1:10 diluted untreated saliva (b, d) were denatured with Laemmli buffer and loaded on a polyacrylamide gel for immunoblotting. The nitrocellulose membrane was blocked in Odyssey blocking buffer (LI-COR Biosciences) for 1 h at room temperature (RT) and washed three times with TBS-Tween. Then the membrane was incubated with goat anti-human IgG coupled with RD680 fluorophore (LI-COR Biosciences, #926-68078) diluted 1:10000 in Intercept T20 antibody diluent (LI-COR Biosciences) for 90 min at RT. The membrane was washed three times, dried and fluorescence emitted at 700 nm was imaged using an Odyssey CLx imaging system (LI-COR Biosciences). The relative quantity of target proteins was estimated by measuring the integrated fluorescence intensity at the corresponding spot after background subtraction using Fiji (ImageJ). Expected bands: 50 kDa (IgG heavy chain), 23.5 kDa (IgG light chain).


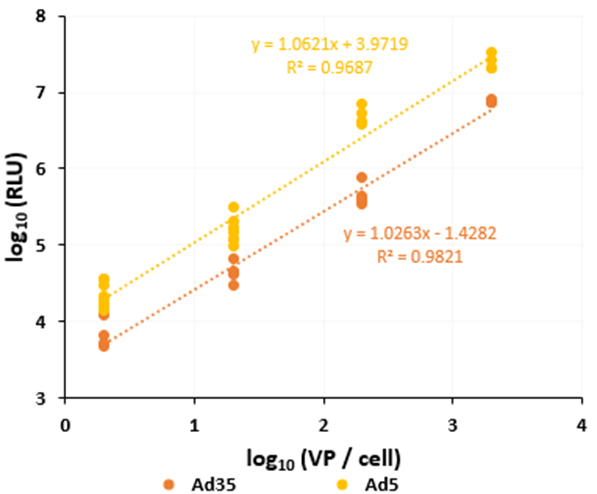


**Supplementary Figure 2. Reporter gene expression level is proportional to infectious dose.** Known doses of reporter-gene expressing Ad5 or Ad35 were used to infect A549 cells and luciferase luminescence was measured at 24 hpi (*n*=5). The logarithms of luminescence levels (RLU = relative luminescence units) and multiplicity of infection (virus particle per cell) were plotted to give equal weight to each infection dose in the statistical analysis. The displayed equations represent the linear regression of logarithmic values. Proportionality was tested in R using the “car” library and the linear Hypothesis function by comparing a non-constrained linear model of logarithms data with a linear model with a slope set to 1, yielding p values of 0.1415 for Ad5 and 0.4457 for Ad35 that showed no significant difference between the proportionality model and the non-constrained regression.
